# Obestatin Enhances In Vitro Generation of Pancreatic Islets through Regulation of Developmental Pathways

**DOI:** 10.1371/journal.pone.0064374

**Published:** 2013-05-31

**Authors:** lessandra Baragli, Cristina Grande, Iacopo Gesmundo, Fabio Settanni, Marina Taliano, Davide Gallo, Eleonora Gargantini, Ezio Ghigo, Riccarda Granata

**Affiliations:** Division of Endocrinology, Diabetology and Metabolism, Department of Medical Sciences, University of Torino, Torino, Italy; University of Santiago de Compostela School of Medicine - CIMUS, Spain

## Abstract

Availability of large amounts of *in vitro* generated β-cells may support replacement therapy in diabetes. However, methods to obtain β-cells from stem/progenitor cells are limited by inefficient endocrine differentiation. We have recently shown that the ghrelin gene product obestatin displays beneficial effects on pancreatic β-cell survival and function. Obestatin prevents β-cell apoptosis, preserves β-cell mass and stimulates insulin secretion *in vitro* and *in vivo,* in both normal and diabetic conditions. In the present study, we investigated whether obestatin may promote *in vitro* β-cell generation from mouse pancreatic islet-derived precursor cells. Treatment of cultured islets of Langerhans with obestatin (i) enriched cells expressing the mesenchymal/neuronal marker nestin, which is associated with pancreatic precursors; (ii) increased cell survival and reduced apoptosis during precursor selection; (iii) promoted the generation of islet-like cell clusters (ICCs) with increased insulin gene expression and C-peptide secretion. Furthermore, obestatin modulated the expression of fibroblast growth factor receptors (FGFRs), Notch receptors and neurogenin 3 (Ngn3) during islet-derived precursor cell selection and endocrine differentiation. These results indicate that obestatin improves the generation of functional β-cells/ICCs *in vitro*, suggesting implications for cell-based replacement therapy in diabetes. Moreover, obestatin may play a role in regulating pathways involved in pancreas development and regeneration.

## Introduction

*In vitro* generation of functional β-cells may help to overcome the shortage of pancreatic islets for replacement therapy in diabetes [1]. To date, insulin-expressing cells have been derived from stem cells (SCs), induced pluripotent stem cells (iPSCs) and precursors obtained from different tissues [1]–[5]. Functional β-cells *in vitro* may be obtained by regulating the expression of genes involved in pancreatic development. This has been attempted through genetic reprogramming and/or growth factor supplementation [1]–[6]. However, despite some success, the use of SCs and genetically reprogrammed cells has raised both ethical and safety issues, whereas growth factor supplementation alone resulted in the generation of β-cells with inefficient insulin synthesis and secretion [1].

*In vivo* β-cell regeneration in adult human and mouse pancreas involving *in situ* precursor cells [7]–[10] suggested that their higher degree of commitment and location would require less manipulation to differentiate into β-cells in *in vitro* settings [1]. Indeed, nestin^+^/vimentin^+^ mesenchymal-like cells (MLCs) have been isolated and enriched from both rodent and human islets [11]–[15]. These are multipotent stromal cells displaying epigenetic marks on the insulin gene, indicating priming for β-cells [11], [13]–[16]. Once expanded, supplementation with specific growth factors or changes in cell culture condition may induce MLC differentiation into hormone expressing islet-like cell clusters (ICCs) containing putative β-cells [11], [13]–[15], [17]. However, ICCs inefficiently release insulin/C-peptide in response to glucose and display poor insulin gene expression [11], [12], [14], [15]. This limitation is only in part overcome with ICC implantation in mice models of diabetes [12]. Thus, although *in vitro* generation of transplantable ICCs from MLCs appears a promising approach to cell replacement therapy, it is still technically challenging.

Obestatin, was discovered as a new ghrelin gene-derived peptide and proposed to bind to the GPR39 orphan receptor and to exert effects opposed to those of acylated ghrelin on food intake [18]. However, both assumptions have been questioned and obestatin physiological role is still quite unknown [19], [20]. Our recent findings suggested that the glucagon-like peptide 1 receptor (GLP-1R) may be involved in at least part of obestatin activities, implying therapeutic potential in metabolic dysfunctions and diabetes [21]. Indeed, obestatin stimulates glucose-induced insulin secretion *in vitro* in β-cell lines and human pancreatic islets [21], and *in vivo* in mice [22] and in perfused rat pancreas [23]. Moreover, like ghrelin [24], obestatin inhibits apoptosis of pancreatic β-cells and human islets, and up-regulates genes essential for β-cell survival and endocrine differentiation [21]. *In vivo* obestatin prevents diabetes in streptozotocin (STZ)-treated rats [25] and reduces insulin resistance in mice fed a high fat diet [22]. Although being mainly produced by the stomach [18], obestatin expression in fetal, neonatal and adult pancreas may imply a role in pancreas development and homeostasis [20], [26]. Based on the foregoing, we hypothesized that obestatin would influence pancreatic precursor differentiation into β-cells. Therefore, we studied obestatin effects on *in vitro* differentiation of mouse pancreatic islet-derived MLCs into functional β-cells/ICCs.

## Materials and Methods

Mouse amidated obestatin (1–23) was from Phoenix Pharmaceuticals (Karlsruhe, Germany). Tetramethyl-6-carboxyrhodamine (TAMRA)-obestatin was from Inbios, Naples, Italy. Cell culture reagents were from GIBCO (Life Technologies, Milan, Italy). Rabbit cytokeratin 19, GLP-1R, ghrelin and somatostatin antibodies were from Santa Cruz Biotechnology (DBA, Milan, Italy). Rabbit GPR39, Oct3/4, PDX1, β-actin antibodies and guinea pig serum insulin antibodies were from Abcam (Cambridge, UK). Alexa Fluor-labeled antibodies were from Molecular Probes (Invitrogen, Milan, Italy). Mouse fibroblast growth factor (FGF2) monoclonal antibody and FGF2-neutralizing antibodies were from Millipore (Milan, Italy). Ngn3 antibody was from Chemicon (Milan, Italy). Primers for RT-PCR were synthesized by Tema Ricerche (Bologna, Italy), All other reagents were from Sigma Aldrich unless specified in the text.

### Animals

Male C57BL6/J mice (6–7 months-old) were used for pancreatic islet isolation. Mouse procedures conformed to Guide for Care and Use of Laboratory Animals of the U.S. National Institute of Health and all procedures were approved by the animal care and use committee of the University of Turin. All mice were double-housed on a 12-h light, 12-h dark cycle (6 am lights on-6 pm lights off) at 22°C and provided standard chow and water ad libitum. Mice were anesthetized with tribromoethylalcohol (Avertin; 375 mg/kg i.p.) and euthanized by cervical dislocation.

### Islet Isolation and Cell Cultures

Pancreases were excised, minced (stage 1) and digested as described [27]. Digestion was quenched by addition of FBS, tissues collected through centrifugation (stage 2), resuspended in RPMI-1640 with 10% FBS and 2 mM glutamine, and plated in 100 mm dishes. Within 3–4 days the exocrine and ductal tissues attached to the dish or died (stage 3). At day 4, 85–95% of floating cell aggregates were positive to dithizone and 99% negative to the ductal marker cytokeratin 19 (Cyto19). Islet yield, over six extractions, was 1142.5±190 islets/mouse. Enriched islets were collected, plated in 6 or 24 multi-well plates coated with 4 µg/ml fibronectin, 1 µg/ml concanavalin A, 0.001% poly-L-ornithin (day 1, stage 4) and maintained in DMEM low glucose (5.5 mmol/L) containing 2 mmol/L glutamine and 10% FBS. ≈45–55 islets were plated in each well of a 6-well plate and 20 in a 24-well plate. Half of the dishes were treated with obestatin (100 nmol/L) every two days. The majority of islets attached to the dish within 3–6 days (stage 4) and no difference in the number of adherent islets was observed upon obestatin treatment. During stage 4 mesenchymal-like cells (MLCs) emerging from the islets were allowed to expand. After 4 days fresh medium was added and at day 6 changed into serum free DMEM/F12 containing 26 mmol/L glucose, 2 mmol/L glutamine, 1400 U/ml LIF, 20 ng/ml FGF2, 20 nmol/L ITS (insulin, selenite, transferrin), 5 µg/ml fibronectin (day 1, stage 5). Cells were never subcultured and medium was replaced twice, with obestatin addition (100 nmol/L). From day 6, with appearance of MLCs tridimensional clusters, fresh medium (300 µl) was added every two days, with obestatin (100 nmol/L), up to day 15 of stage 5. All experiments were performed the day after obestatin treatment.

### Islet Counting and Morphometric Analysis

Islets were stained with dithizone for 20 min at 37°C. Red cell clusters were counted as islets in 10 random fields. Microphotographs were taken at fixed time intervals (10X). Islet area was evaluated using Image J software (http://rsbweb.nih.gov/ij/) using a Leica DM200 microscope and a Leica DFC340 FX camera. Observations were repeated in 6 different isolations. Dithizone stained islets were not used for subsequent experiments.

### Insulin and C-peptide Secretion

5 islets or ICCs were hand-picked under the microscope and incubated with different concentrations of D-glucose in Krebs-Ringer buffer to induce insulin/C-peptide secretion [21]. L-glucose, was used as molar control. Islets were collected, supernatants stored at −20°C and tested for insulin and C-peptide (mouse RIA and ELISA kit, respectively, Alpco Diagnostics, DiaSorin, Saluggia, Italy). Insulin was also tested in medium alone (300 µl).

### Cell Migration

Cell migration was assayed in Boyden’s chambers. Cells were FGF2-starved for 12 h, detached in PBS/0.5% EDTA and resuspended in FGF2-deprived serum-free medium. 125,000 cells were seeded on 0.1% gelatine coated filters (8-µm pore size, NeuroProbe, DBA, Milan, Italy) and incubated at 37°C for 4 h. The lower compartment contained serum-free medium either FGF2-deprived, with 100 ng/ml FGF2 or 100 ng/ml FGF2 and 2.5 µg/ml FGF2-neutralizing antibody. Trapped-cells in the filter were fixed in cold methanol and stained with 2% Giemsa, then counted in four different fields under a light microscope (Leika DMIL).

### RT-PCR and Real-time PCR

RNA extraction and reverse transcription were performed as described [21]. For RT-PCR experiments, cDNA (9 µl) was amplified (GeneAmp PCR System; Perkin Elmer, Milan, Italy) in 50 µl (94°C for 30 s, for 64°C for 30 s; 72°C for 30 s, 72°C for 7 min.) For semiquantitative analysis, we optimized cDNA amount and determined the optimal cycle number in the linear range of PCR amplification for each gene. PCR products were separated by 2% agarose gel electrophoresis and visualized by ethidium bromide staining. For real-time PCR, cDNA was treated with DNA-free DNase (LifeTech, Monza, Italy). Real-time PCR was performed with 50 ng cDNA, 150 nmol/L of each primer and the IQ-SYBR-green mastermix (BioRad, Milan, Italy) using the ABI-Prism 7300 (Applied Biosystems). The expected fragment sizes, conditions and primers are reported in Table S1.

### Apoptosis

Morphological changes in the nuclei of apoptotic cells were detected by Hoechst 33258, as described [21].

### Cell Survival and Dimension

Cell survival was assessed as described [22]. Cells were detached from dishes with 0.05% trypsin/EDTA and stained with Trypan blue dye (0.04% w/v; Invitrogen). Cells were counted with a Countess automated cell counter (Invitrogen). Cell dimension was automatically calculated during this procedure and expressed in µm.

### Immunofluorescence Analysis

For differentiation markers, islets/cells were grown on coverslips, fixed in ice-cold 4% paraformaldehyde (PFA) and processed as described [22]. Antibodies used: rabbit polycolonal antibody for nestin (1∶100), Oct3/4 (1∶80), Ngn3 (1∶100), PDX1 (1∶800), glucagon (1∶100), somatostatin (1∶100), ghrelin (1∶100), rhodamine-labeled anti-ghrelin and anti-somatostatin (both 1∶100); guinea pig serum anti-insulin (1∶80); Alexa-488-conjugated antibodies (1∶450). For cell proliferation, BrdU (10 µM) was added to cells at day 3 of stage 4 and at day 6 of stage 5. After 72 h cells were fixed, treated with 2 mol/L HCl for 45 min at room temperature, neutralized with 0.1 mol/L sodium borate (pH 8.5) for 15 min and processed for staining. Alexa-546 labeled anti-BrdU antibodies (1∶50) were used to assess BrdU incorporation. BrdU positive cells and nuclei were counted (100 to 500 cells) in 20 microscopic fields (X20). TAMRA-obestatin (TAMRA-Obe), was used to visualize obestatin binding in adherent islets and MLCs on coverslips as described [22]. For GPR39 and GLP-1R, cells were permeabilized with 0.2% Triton X-100 after fixation, blocked in 10% goat serum, stained overnight with rabbit polyclonal anti-GPR39 (1∶150) or anti-GLP-1R (1∶100) and incubated 1 h at room temperature with Alexa-488-conjugated goat anti-rabbit antibody (1∶450). Nuclei were stained with DAPI. The same procedure was followed for TAMRA-obestatin co-localization with nestin. Images were taken using a Leica DM200 fluorescent microscope and a Leica DFC340 FX camera. For FGF2, cultures were FGF2-starved for 2 h or not, fixed in ice-cold4%PFA, then treated as described [22]. Anti-FGF2 was used at 1∶100 for 2 h at room temperature. 100 to 500 nuclei were counted in 10 microscopic fields.

### Western Blotting

Immunoblot analysis were performed as described [22]. Anti-nestin antibody was used at 1∶500. Blots were reprobed with β-actin (1∶2000) for normalization. Immunoreactive proteins were visualized with Chemidoc XRS (Bio-Rad, Milan, Italy), and densitometric analysis performed with Quantity One software (Bio-Rad).

### Glucose Levels and Protein Content

Glucose was measured in cell conditioned medium (300 µl). Samples were frozen at −80 C and quantified with the GLUC-PAP kit (Menarini, Florence, Italy), according to manufacturer’s instructions. Protein concentrations were determined with BCA Protein Assay Kit (Thermo Scientific, Pierce, Milan, Italy).

### Statistical Analysis

Data are presented as means ± SEM. Results were analyzed using 2-tailed Student’s t test or two-way ANOVA followed by Tukey’s HSD for post-ANOVA comparisons (GraphPad Prism 5.0 software, San Diego, CA). Significance was established when *P*<0.05.

## Results

### Obestatin Influences ICC Number and Size

**Figure 1:**
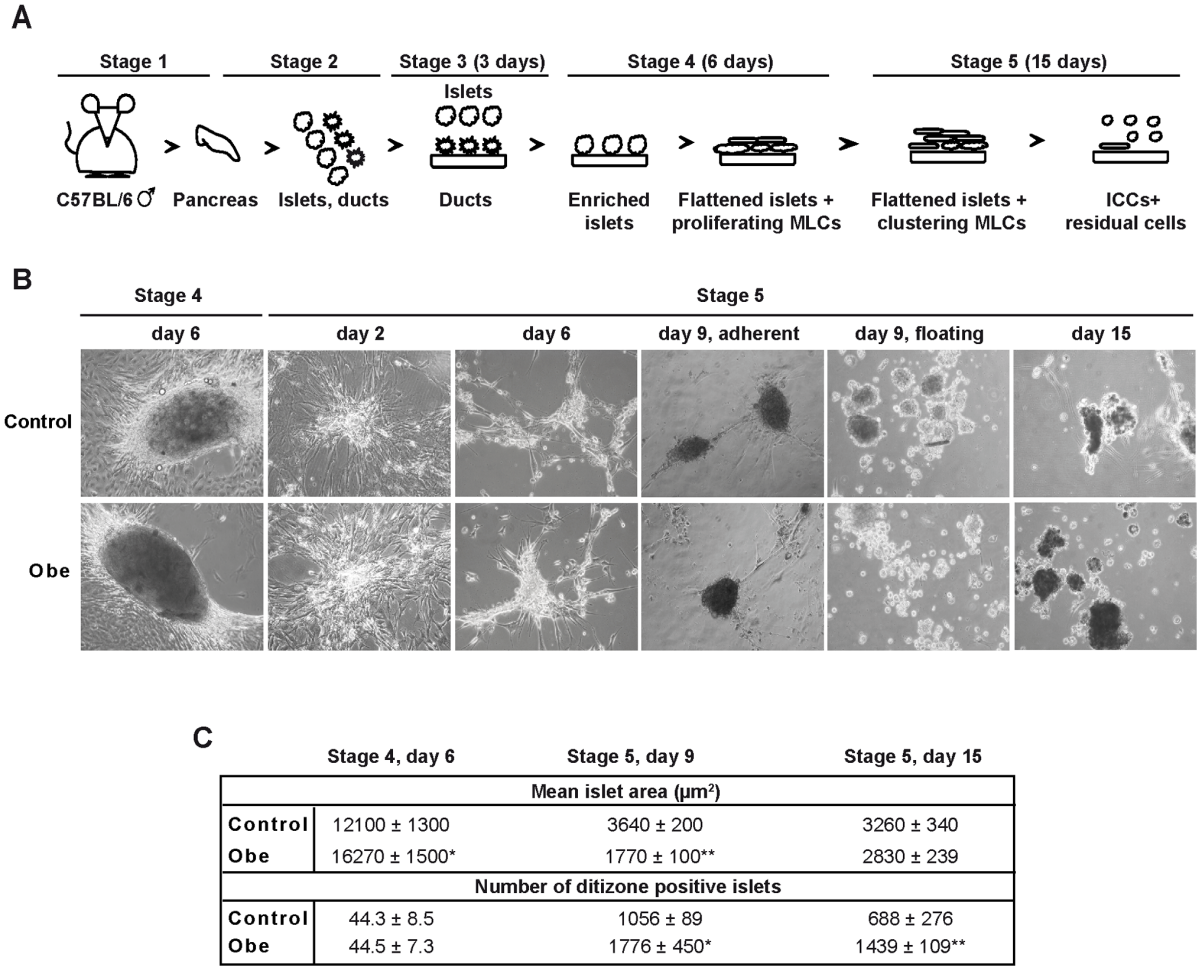
Overview of culture evolution. For details also see Materials and Methods. (A) Schematic representation of the protocol used to obtain ICCs from mouse pancreas. Stage 1, pancreas removal; stage 2, pancreas digestion and plating; stage 3, islet enrichment; stage 4, islet adhesion and MLC appearance; stage 5, MLC migration, clustering and ICC formation. (B) Representative photomicrographs comparing the evolution of control and obestatin-treated (Obe) cultures. Islet adhesion to the plate induced generation of MLCs (stage 4). During stage 5 MLCs migrate and assemble to form ICCs. Magnification, 10X. (C) Mean surface area and number of adherent islets (stage 4 day 6) and floating ICCs (stage 5) in either control or obestatin treated conditions. Results are the mean ± SEM. *P<0.05, **P<0.01 vs. control; ^##^ *P*<0.01 vs. same condition at stage 4, day 6. N = 6.

Pancreatic islets were isolated and enriched (stage 1–3), then cultured for 6 days in serum-containing medium (stage 4), in either absence or presence of obestatin (Fig. 1*A and B*). Most of dithizone^+^ islets attached to the plate, flattened and generated rapidly proliferating MLCs (Fig. 1A and B). At day 6 (stage 4) obestatin-treated adherent islets displayed increased area with respect to control islets (Fig. 1C). At this stage, both untreated and obestatin-treated islets showed expression of GPR39 and GLP-1R, as assessed by RT-PCR and immunofluorescent staining (Fig. S1).

The change to serum-free medium (stage 5) caused MLC migration away from the original islet. By day 6 (stage 5) they initiated aggregation into tridimensional structures (Fig. 1A and B) and at day 9 both adherent MLCs and early floating ICCs were observed (Fig. 1B). Most of MLCs assembled into ICCs by day 15 (Fig. 1A and B). At day 9 (stage 5), obestatin treatment reduced ICC area, whereas increased ICC number, with respect to control (Fig. 1C). At day 15 ICC area was comparable between control and obestatin-treated, although obestatin still increased ICC number (Fig. 1C). Compared to adherent islets (stage 4, day 6) ICC size was strongly reduced, whereas ICC number increased, in both obestatin-treated or control (Fig. 1C).

### Obestatin Increases Insulin and Glucagon Expression in ICCs

**Figure 2:**
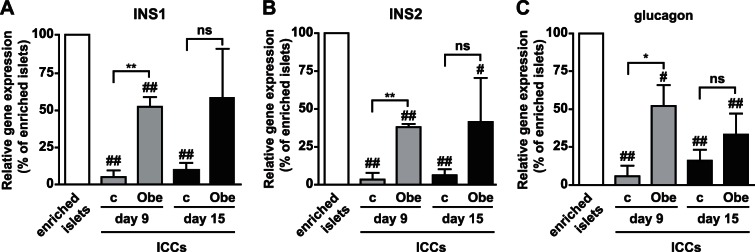
Obestatin up-regulates insulin and glucagon gene expression. INS1 (A), INS2 (B) and glucagon (C) mRNA expression in enriched islets and ICCs at day 9 and day 15 (stage 5) in control (c) and obestatin-treated (Obe) cultures evaluated by Real Time PCR. Gene expression was normalized to β-actin and reported as percent of enriched islets. *P<0.05, **P<0.01 vs. c; ^#^ *P*<0.05, ^##^ *P*<0.01 vs. enriched islets. N = 4 (ns, not significant).

**Figure 3:**
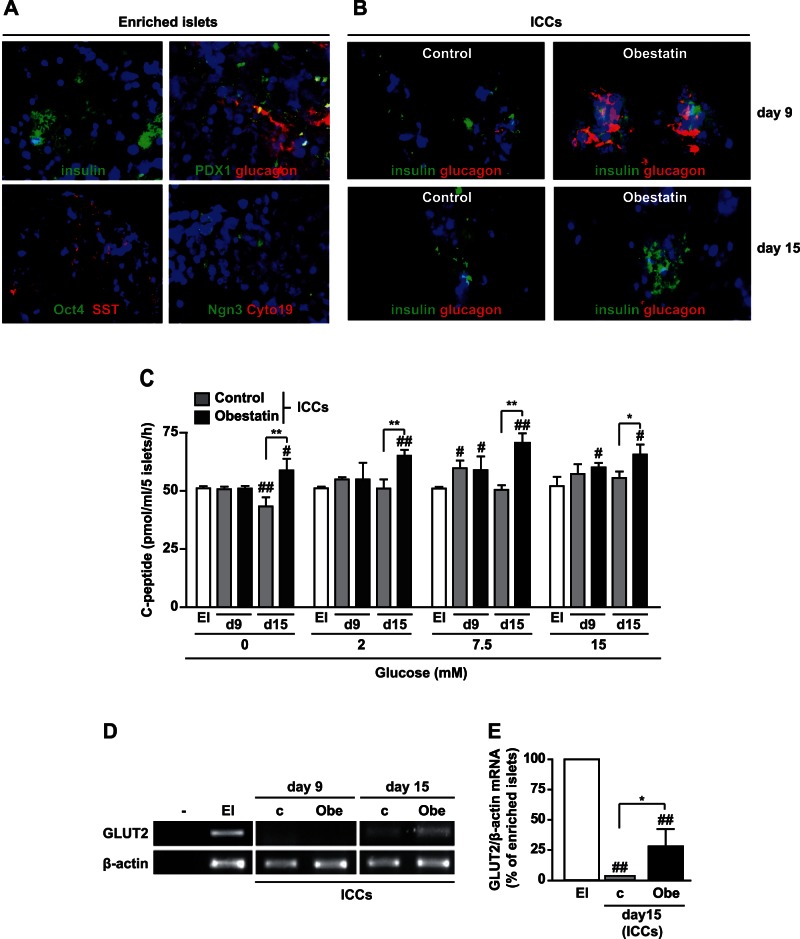
Differentiation markers and hormone expression in islets and ICCs and obestatin effects on C-peptide secretion. (A) Fluorescence microscopy images showing enriched islets stained for insulin, glucagon and somatostatin and the indicated markers of progenitors (magnification, X20). Cytokeratin 19 (Cyto19) was used as marker for pancreatic ducts. (B) Immunofluorescent staining for insulin and glucagon in ICCs formed in either absence (control) or presence of obestatin at the indicated days (magnification, X40). (C) C-peptide secretion, assessed by ELISA, from ICCs stimulated at either day 9 or day 15 with the indicated concentrations of glucose. Results are the mean ± SEM of three different experiments performed in duplicate (*P<0.05, **P<0.01 vs. control ICCs; ^#^ *P*<0.05 ^##^ *P*<0.01 vs. enriched islets, EI). (D) GLUT-2 mRNA determined by RT-PCR. β-actin was used as housekeeping gene. Shown is a representative image of three independent experiments. Buffer alone was used as negative control (–) (c, control, no obestatin). (E) Densitometric analysis of GLUT-2 mRNA normalized to β-actin and reported as percent of enriched islets (EI) (*P<0.05 vs. control ICCs; ^##^ *P*<0.01 vs. EI).

Obestatin effects were investigated on insulin and glucagon mRNA expression in ICCs, at day 9 and 15 (stage 5). ICCs displayed lower insulin (INS1, INS2) and glucagon at either day 9 or 15 as compared to enriched islets. Notably, obestatin up-regulated INS1, INS2 and glucagon mRNA at day 9 with respect to control, but not at day 15 (Fig. 2A–C). Enriched islets, ICCs at day 9, but also at day 15, were positive to insulin and glucagon also at protein level (Fig. 3A and B). Obestatin-treated ICCs displayed enhanced insulin staining at day 9 and 15 and greater glucagon expression at day 9 compared to control. At day 15, instead, glucagon was barely detectable in both conditions (Fig. 3B). Enriched islets were positive for somatostatin (SST) at protein level (Fig. 3A). Conversely, no gene and protein expression for SST in ICCs, and for ghrelin in enriched islets and ICCs, was detected. Enriched islets also expressed PDX1, a β-cell progenitor marker [3], [6], [11] (Fig. 3A). Few cells in the islets expressed neurogenin 3 (Ngn3), marker of endocrine-committed pancreatic progenitors [28], but none expressed the stem cell marker octamer-binding transcription factor 4 (Oct4) [3] (Fig. 3A). Furthermore, islets were negative for the pancreatic duct marker cytokeratin 19 (Cyto19) [8], suggesting absence of duct contamination (Fig. 3A).

### Obestatin Enhances Glucose-stimulated C-peptide Secretion in ICCs

Cells grown in insulin-containing medium may take-up and release exogenous insulin or display insulin immunoreactivity without real endocrine differentiation [29]. Therefore, glucose-stimulated insulin secretion was determined (Fig. S2A) and compared to insulin levels in cell culture medium (Fig. S2B). Obestatin-treated ICCs displayed the highest response at day 9, followed by control ICCs (Fig. S2A),whereas control ICCs at day 15 showed the lowest insulin secretion (Fig. S2A). In culture medium, insulin levels were sustained until day 6, indicating additional insulin secretion from residual β-cells, but decreased abruptly at day 9 when ICCs had formed, confirming uptake of exogenous insulin (Fig. S2B). Nonetheless, the difference in insulin release between control and obestatin-treated ICCs suggest *de novo* insulin synthesis, in line with the mRNA expression data (Fig. 2A and B).

To confirm *de novo* insulin synthesis, C-peptide secretion [29], [30] was measured in enriched islets and ICCs, either untreated or treated with obestatin. Basal C-peptide release in enriched islets was unchanged upon glucose-stimulated conditions, likely because of the strict extraction procedures (Fig. 3C). At day 9, control and obestatin-treated ICCs displayed comparable secretion. However, at day 15, obestatin strongly increased C-peptide release compared to control at all glucose concentrations (Fig. 3C), except at 25 mmol/L where glucose was likely cytotoxic (data not shown). In line with improved endocrine differentiation and/or glucose sensitivity, the β-cell glucose transporter 2 (GLUT2) mRNA was increased at day 15 compared to control in obestatin-treated ICCs, being barely detectable at day 9. Conversely, GLUT2 was strongly expressed in enriched islets (Fig. 3D and E).

### Obestatin Promotes the Increase of Nestin^+^ Cells in Enriched Islets

To elucidate the cellular/biological events underlying obestatin-induced improvement of ICCs endocrine differentiation, we assessed proliferation of cells expressing the different hormones and differentiation markers [3], [6], [11], [28] at day 6 of stage 4, before the progenitor selection phase. These included nestin, Oct4, PDX1 and Ngn3. Within the islet, nestin^+^ cells were the prevalent population upon adhesion, independently of obestatin treatment (Table 1). Obestatin increased glucagon^+^ cell number but decreased their proliferation, likely improving commitment of cells to the alpha type. Obestatin did not affect Ngn3^+^ cell proliferation, although reduced their percentage, suggesting differentiation into other cell types [28], [31]–[33]. Conversely, obestatin increased insulin^+^ cell number and proliferation, suppressed somatostatin expression and prevented PDX1^+^ and Oct4^+^ cell proliferation (Table 1).

**Table 1:** Enumeration of endocrine hormone-producing cells and BrdU^+^ cells within the islet (day 6, stage 4).

Total cells (%)			BrdU positive cells (%)	
Control		Obestatin	Control	Obestatin
Insulin	3.5±1.9	18.8±6.9*	51.5±1.5	57.4±2.6*
Glucagon	31.9±4.5	40.5±2	36.6±7	14.1±5.1*
Ghrelin	3.9±2.2	1.8±0.2	79.2±12	78.4±12.5
Somatostatin	1.3±0.8	0±0	76.45±13.6	0±0**
PDX1	48.9±9.4	48.3±13.2	24.9±0.6	8±3.6**
Ngn3	26.2±9.1	10.9±3.4*	45.1±14.6	41.5±12.5
Nestin	65.4±11	59.2±7.6	18.2±7.5	8.4±8.4
Oct4	7.2±1.3	5.7±1.3	58.4±4.7	2.5±2.5**

Results are the mean ± SEM of three independent experiments (*P<0.05; **P<0.01; n = 3).

Obestatin promoted late proliferation of few insulin^+^ cells in MLCs (Table 2) and mildly increased ghrelin^+^ cell number. It also caused disappearance of Ngn3^+^ cells. Most of all, obestatin strongly increased nestin^+^ MLCs, that likely co-expressed other markers (Table 2) [3], [13], [34], although decreasing their late proliferation (Table 2). Despite these effects, obestatin did not influence total cell proliferation (15.3±1.8 BrdU^+^ cells in control islets vs. 15.2±0.1% in obestatin-treated islets, 14.2±2.2% BrdU^+^ cells in control MLCs vs. 14±4.3% in obestatin-treated MLCs) and cell dimension (control, 14.9±1.39 µm; obestatin, 16.2±0.9 µm). These results confirm previously described mitogenic effects on β-cells [21] and suggest that obestatin enriches nestin^+^ MLCs and negatively modulates Ngn3 expression at stage 4.

**Table 2:** Enumeration of endocrine hormone-producing cells and BrdU^+^ cells (proliferating MLCs; day 6, stage 4).

Total cells (%)			BrdU positive cells (%)	
Control		Obestatin	Control	Obestatin
Insulin	5.4±2	5.5±3	18.9±10.6	53.7±7.5*
Glucagon	12.8±3.4	11.1±5	3.4±2.3	0±0
Ghrelin	0.27±0.27	3.1±0**	0±0	0±0
Somatostatin	0.4±0.4	2.4±2.4	0±0	4±4
PDX1	13±3.7	11.4±3.6	12.2±7	9.3±4
Ngn3	15±10.6	0±0**	48.5±2.3	0±0**
Nestin	20.6±8.8	97.5±2.5**	52.5±14	21.9±0.7*
Oct4	12.8±6	2.8±2.39	21.6±14.1	10±10

Results are the mean ± SEM of three independent experiments (*P<0.05; **P<0.01; n = 3).

### Obestatin Binds to Adherent Islets, MLCs and Neuronal-like Nestin^+^ cells at the End of Stage 4

**Figure 4:**
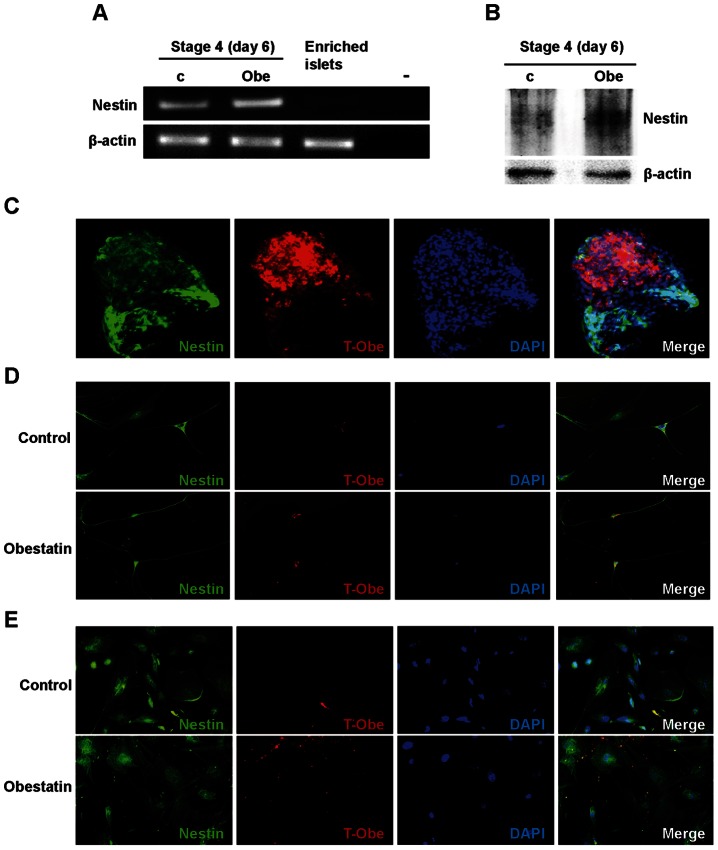
Obestatin up-regulates nestin expression at stage 4 and binds to enriched islets and MLCs. (A) Nestin mRNA assessed by RT-PCR in control (c) and obestatin-treated cells (Obe). Buffer alone was used as negative control (–). Shown is a representative image of three independent experiments. (B) Nestin protein expression determined by Western blot in lysates cultures that were either untreated or treated with obestatin (n = 3). (C) TAMRA-Obestatin (T-Obe) (1 µM) binding to adherent islets and emerging MLCs. The image is representative of both untreated and obestatin-treated cultures, that were tested in at least three independent experiments (magnification, X20). (D-E) T-Obe (1 µM) binding to nestin^+^ neuronal-like cells (D) and to Nestin^+^ MLCs (E) in both control and obestatin-treated cultures (n = 3; magnification, X40). T-Obe is shown in red, nestin in green and nuclei in blue (DAPI).

In line with the increased percentage of nestin^+^ MLCs (Table 2), obestatin up-regulated nestin gene expression and protein at the end of stage 4 (Fig. 4A and B). Therefore, we investigated whether obestatin may directly bind to MLCs and islets. At day 6 of stage 4, TAMRA-obestatin (T-Obe) stained adherent islets and also the soma of nestin^+^ neuronal-like cells, in both control and obestatin-treated cultures (Fig. 4C and D). T-Obe binding was observed in the majority of obestatin-treated nestin^+^ MLCs, compared to control MLCs (Fig. 4E).

### Obestatin Reduces Apoptosis during ICC Formation at Stage 5

**Figure 5:**
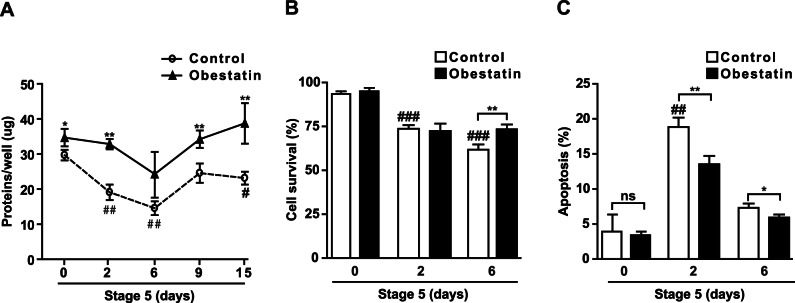
Obestatin promotes cell survival and decreases serum starvation-induced apoptosis at stage 5. (A) Protein content in control and obestatin-treated cultures at the indicated days. (B-C) Cell survival (B) and apoptosis (C) assessed by Trypan blue exclusion and Hoechst 33258 staining, respectively, at the indicated days. For all graphs, results are the mean ± SEM of three independent experiments (*P<0.05, **P<0.01 vs. control; ^#^ *P*<0.05, ^##^ *P*<0.01, ^###^ *P*<0.001 vs. day 0).

Serum-free medium switch at the end of serum-stimulated cell growth was shown to cause apoptosis selection and expansion of pancreatic progenitors [11], [15], [35], [36]. We monitored cell survival and apoptosis at day 2 and 6 (stage 5) when most cells were still adherent. Protein content in control cultures dramatically decreased at day 2 and 6 of stage 5, returning to values close to day 0 (i.e. day 6 of stage 4) only from day 9 (Fig. 5A). Conversely, in obestatin-treated cultures, protein content was unchanged with respect to day 0, suggesting increased cell viability (Fig. 5A). Cell survival was reduced at day 2 and even more at day 6 as compared to day 0 (Fig. 5B) and was increased by obestatin at day 6 (Fig. 5B). Obestatin inhibited apoptosis at day 2 compared to control (Fig. 5C). At day 6, apoptosis was halved in control cells, suggesting that cell selection was complete [11]; however, obestatin still displayed antiapoptotic actions (Fig. 5C).

### Obestatin Reduces FGF2-induced Cell Migration of MLCs at Stage 5

**Figure 6:**
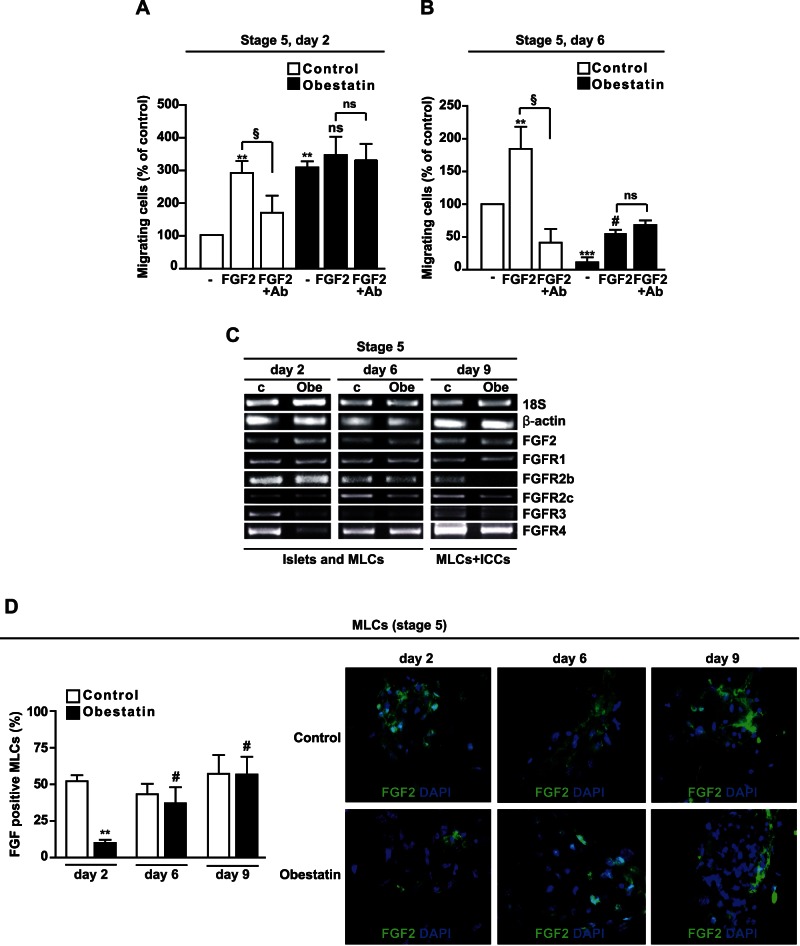
Obestatin inhibits cell migration and FGF2 binding to MLCs at stage 5. (A–B) Basal and FGF2-induced cell migration assessed at day 2 (A) and 6 (B) in either untreated cells (Control) or obestatin-treated cultures (–, no FGF2). Neutralizing antibody against FGF2 (Ab) was used to verify the specificity of FGF2 effect. The cells were deprived of FGF2 for 12 h then exposed for 4 h to 100 ng/ml of FGF2 and 100 ng/ml of neutralizing antibody. Results are the mean ± SEM of three independent experiments performed in duplicate (**P<0.01, ***P<0.001 vs. control; ^#^ *P*<0.05 vs. –; ^§^ *P*<0.05, ^§§^ *P*<0.01; ns, not significant). (C) Expression of FGF2 and FGFRs assessed by RT-PCR at the indicated days in control (c) and obestatin-treated cultures (Obe). Results are representative of three independent experiments. (D) FGF2^+^ MLCs that were cultured in non FGF2-starved conditions, in either absence or presence of obestatin at the indicated days. Results shown in the graph (left) are expressed as percent of total cell number and are the mean ± SEM of three independent experiments performed in duplicate (**P<0.01 vs. control, ^#^ *P*<0.01 vs. obestatin at day 2). Right panel, representative images (X20) of FGF2 staining (green) (n = 3); nuclei are shown in blue (DAPI).

At stage 5, FGF2 in the medium is initially required for selection of pancreatic progenitors [11], [15], [35], [36] and later, to initiate their endocrine differentiation [37]. FGF2 also plays a role in precursor cell migration and aggregation into ICCs [37]. We investigated FGF2 effect on cell migration in obestatin-treated MLCs at day 2 and 6, when most cells were still adherent. At day 2, FGF2 strongly increased cell migration in cells cultured in the absence of obestatin (control). This effect was inhibited by an FGF2-neutralizing antibody, suggesting specificity (Fig. 6A). Conversely, obestatin strongly increased MLC basal migration at day 2, but no additional effect was displayed by FGF2 and its neutralizing antibody (Fig. 6A). At day 6, the FGF2 stimulatory effect on control cell migration was specific and similar to day 2 (Fig. 6B). Instead, obestatin-treated cultures showed dramatically reduced basal migration compared to control cells. Moreover, although FGF2 increased migration, the neutralizing antibody was ineffective, suggesting non specific FGF2 action (Fig. 6B). The different results obtained at day 2 and 6 were independent of cell size, which was similar in control and obestatin-treated cells (day 2, control: 18.6±1.3 µm, obestatin: 20.8±1.7 µm; day 6, control: 19.2±0.6 µm, obestatin: 17.4±1 µm).

### Obestatin Reduces FGF Receptor Expression and FGF2 Binding at Stage 5

To further understand obestatin effects on FGF2 altered sensitivity, we evaluated the expression of FGF2 and its receptors (FGFRs). Both control and obestatin-treated cultures showed comparable FGF2 gene expression at day 2, 6 and 9 (Fig. 6C), in agreement with endogenous FGF2 immunoreactivity in FGF2-starved cells. Conversely, at day 2 obestatin down-regulated FGFR3 and FGFR4 mRNA compared to control (Fig. 6C). At day 6, FGFR mRNA levels in control and obestatin-treated cultures were similar, except for FGFR2c that was slightly down-regulated by obestatin. At day 9, obestatin-treated cultures displayed reduced FGFR2b, FGFR2c and FGFR4 expression compared to control (Fig. 6C), in line with endocrine differentiation [37]. No change over time and treatment was detected for FGFR1 mRNA (Fig. 6C). FGFR gene expression decrease may in part be involved in the different migratory profile of obestatin-treated cells (Fig. 6A and B). To determine this, we compared control and obestatin-treated MLC ability to bind to FGF2. FGF2 immunoreactivity was assessed in cells cultured in normal conditions (FGF2-containing medium). At day 2, FGF2 positivity was strongly reduced in obestatin-treated cells compared to control, whereas at day 6 and at day 9, it was similar between control and obestatin-treated MLCs (Fig. 6D). Similar results were obtained in FGF-starved conditions (Fig. S3). The apparent discrepancy between receptor expression in the whole culture (Fig. 6C) and of FGF2 binding in obestatin-treated MLCs at day 9 (Fig. 6D), may depend on the presence of differentiated ICCs in obestatin-treated cultures. Indeed, at the periphery of obestatin-treated MLC clusters and in the central ICC-forming region, FGF2 binding was decreased with respect to control MLCs (Fig. 6D and S3).

### Obestatin Down-regulates Notch Receptor Gene and Up-regulates Ngn3 Gene and Protein Expression at Stage 5

**Figure 7:**
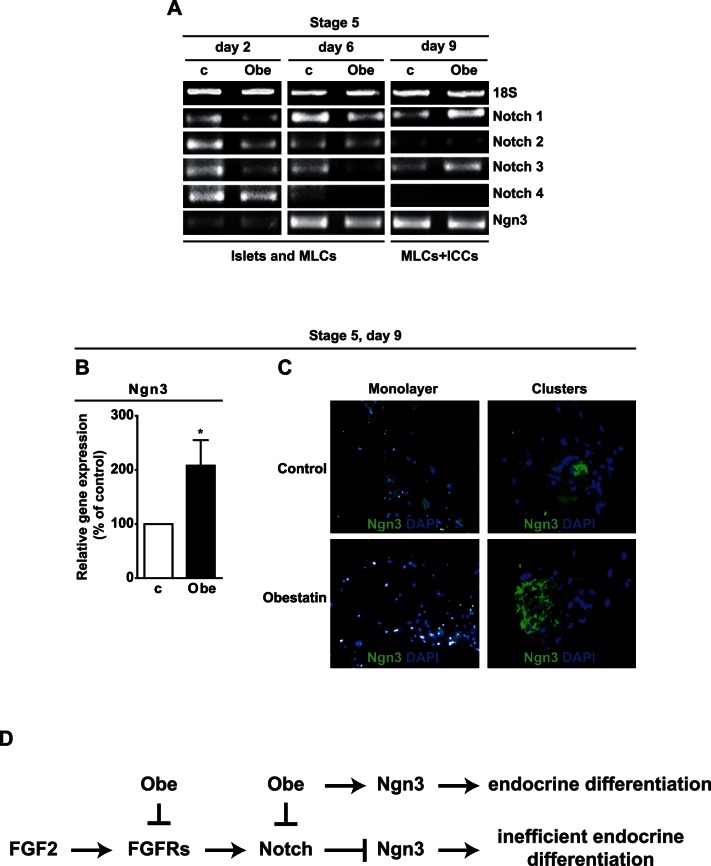
Obestatin modulates Notch receptors and Ngn3 gene expression. (A) RT-PCR analysis showing Notch receptors and Ngn3 mRNA at the indicated times in control (c) and obestatin-treated (Obe) cultures. Results are representative of three independent experiments. (B) Ngn3 mRNA assessed by real-time PCR at day 9 of stage 5 in adherent MLCs. Results are the mean ± SEM of three independent experiments performed in duplicate (*P<0.05 vs. control). (C) Immunofluorescent staining for Ngn3 in adherent cells (monolayer) (X20) and clustered cells (X40), in both control and obestatin-treated cultures. Ngn3 is shown in green and nuclei in blue (DAPI). Each image is representative of three independent experiments. (D) Schematic illustration of obestatin mechanisms of action. During pancreatic development FGF2 promotes pancreatic precursor cell proliferation through FGFRs, inducing Notch signaling and preventing Ngn3 expression. Upon serum removal, FGF2 promotes MLC proliferation and Notch receptors expression. Obestatin treatment inhibits FGF2 action by reducing expression of FGFRs. Obestatin also inhibits the expression of selective Notch receptors, leading to increased Ngn3 expression and endocrine differentiation.

FGFRs stimulation in pancreatic precursor cells maintains active Notch signaling and inhibits Ngn3 expression and endocrine differentiation [28], [38]. While data on Notch ligands Jagged 1,2, Delta-like 1, 3 and 4 expression were inconsistent, expression of Notch receptors varied during stage 5 (Fig. 7A). Obestatin reduced Notch 1, 2 and 3 expression at day 2, and Notch 1, 3 and 4 at day 6. Conversely, it increased Notch 1 and 3 at day 9 (Fig. 7A). Ngn3 gene expression increased at day 6 with respect to day 2 in both control and obestatin-treated cultures, suggesting MLC commitment toward endocrine differentiation (Fig. 7A). As Ngn3 decreases at the end of differentiation, we hypothesized that inclusion of ICCs in gene expression analysis at day 9 would mask Ngn3 differential expression in control and obestatin-treated MLCs. Indeed, we found that Ngn3 in obestatin-treated MLCs at day 9 was increased vs. control (Fig. 7B). Moreover, immunofluorescence studies showed that, whereas in control cultures Ngn3 positivity was low and mainly localized outside the nuclei, in obestatin-treated MLCs Ngn3 was high and mainly nuclear (Fig. 7C, monolayer). Moreover, Ngn3 expression in cells acquiring tridimensional shape was greater in obestatin-treated MLCs compared to control (Fig. 7C, clustered). These results suggest that obestatin modulates Notch receptors and up-regulates Ngn3 gene and protein in MLCs during ICC formation. Table S2 shows the percentage and proliferation rate of MLCs expressing the different hormones and differentiation markers at day 9 of stage 5. Residual MLCs on the plate were either sparse or clustering to form additional ICCs. All hormones and markers were highly expressed, especially glucagon, but not insulin, in line with appearance of β-cells following that of α-cells [28], [39]. Obestatin also decreased somatostatin^+^ cell number and proliferation and inhibited Ngn3^+^ cell proliferation, suggesting commitment of Ngn3^+^ cells toward differentiation (Table S2).

## Discussion

This study shows obestatin effects on *in vitro* generation of ICCs from islet-derived MLCs. Insulin and glucagon gene expression and glucose-stimulated C-peptide secretion were increased in obestatin-treated ICCs as compared to control. Improved *in vitro* ICC endocrine differentiation may be explained by obestatin-induced down-regulation of FGFRs, selective modulation of Notch receptors and timely induction of Ngn3.

Pancreatic β-cells obtained from human and rodent islet-derived pancreatic progenitors have shown deficient insulin synthesis and glucose-induced insulin/C-peptide secretion [1], [11], [12], [14], [15], [40]. These limits have been recently improved by supplementation with growth factors involved in pancreas regeneration and function [3]–[5], [7], [9]. Here, obestatin increased insulin gene expression in ICCs at day 9 to 30–50% of enriched islets, a remarkable improvement compared to control ICCs and to previous findings [1], [11]–[15]. Furthermore, glucose dependent C-peptide secretion and enhanced GLUT-2 expression in obestatin-treated ICCs suggested efficient MLC differentiation into β-cells at day 15. Generation of more functional ICCs involves obestatin binding to adherent islets and nestin^+^ MLCs, enrichment of nestin^+^ MLCs, reduction of apoptosis and increased MLC survival. Thus, mainly in nestin^+^ MLCs, obestatin might stimulate endocrine differentiation.

Besides obestatin, another major difference from previous protocols [11], [14], [15] is that here the cells were never detached from the original plate, to avoid cell death and quiescence. The older age of mice (six/seven months) may explain this reduced cell viability and β-cell proliferation [41]. Moreover, the reservoir of non differentiated cells is likely restricted to those eventually implicated in pancreatic regeneration [7], [8], [10], [42] and β-cell survival [10]. Here, our cell separation-free protocol, where MLCs were maintained with their ‘mother’ islets, may have better mimicked the *in vivo* condition, being less harmful to residual precursors [7], [8], [10], [43]. This culture method may not provide a renewable supply of ICCs; however, previous studies demonstrated that ICCs may be replated to generate novel MLCs and ICCs [13]. Additional studies will clarify whether obestatin-treated ICCs display increased survival and growth, and achieve the functional complexity of an islet once transplanted *in vivo* (4, 5). Our findings may help improving the current protocols for *in vitro* β-cell regeneration and suggest that endogenous/exogenous obestatin influences pancreas regeneration and development. Interestingly, enriched islets expressed here PDX1 and Ngn3, both involved in pancreas regeneration [7], [42].

*In vivo*, blockade of FGF-FGFRs signaling causes disruption of precursor cell proliferation, untimely endocrine differentiation and impaired pancreas organogenesis [35], [38], [44], [45]. Obestatin selectively reduced FGFR expression. Moreover, obestatin altered cell response to FGF2, a mitogen for nestin^+^ mesenchymal stem cells and pancreatic progenitors [15], [35], [36] and chemoattractant for pancreatic progenitors [37], such that FGF2 failed to induce migration in obestatin-treated cells. This occurred at day 2 when FGFR3 and FGFR4 were down-regulated, and at day 6, when FGF2 binding and FGFR expression were similar to untreated MLCs.

Here, we found that FGF2 migratory effects were blocked by a specific anti-FGF antibody in control but not in obestatin treated cells. This may be interpreted as i) an FGF2-independent migration of obestatin treated cells at day 2, when the expression of FGFR specific isoforms was reduced, ii) a partial recovery of FGF2-induced migration in obestatin treated cells at day 6, when expression and binding of FGFRs returned similar to control cells. In both cases, the lack of effect of the neutralizing antibody in obestatin treated cells may be due to the existing difference in the pool of receptor-associated/modulating proteins with respect to control, such that formation of an FGF2-antibody complex may not prevent binding of FGF2 to its receptor.

As inhibition of FGFR2b and FGFR2c, the most FGF2-sensitive isoforms, has been associated to incorrect pancreas organogenesis and early endocrine differentiation [37], [45], [46], obestatin-induced down-regulation of FGFR2b and FGFR2c at day 9 may relate to advanced endocrine differentiation. Moreover, the small obestatin-induced FGFR2c down-regulation at day 6 suggests that endocrine differentiation starts earlier, in line with decreased MLC basal migration.

FGFR3 blockade *in vivo* causes increased proliferation of immature pancreatic epithelium and islet precursors [47]. Conversely, FGFR4 is expressed in embryonic pancreatic rudiments, characterized by high proliferation of precursors [35]. FGFR3 down-regulation by obestatin at day 2 may be linked to increased proliferation/decreased apoptosis of MLCs. Moreover, obestatin-induced FGFR4 down-regulation may relate to reduced proliferation (day 2) and improved differentiation (day 2 and 9) of committed cells.

FGFR stimulates Notch signaling, which inhibits Ngn3 expression, preventing untimely endocrine differentiation of pancreatic progenitors and allowing their expansion during pancreas development [38]. In obestatin-treated cultures, Notch 2 and 4 down-regulation at day 2 and 6 may follow reduced FGFRs signaling at day 2. Obestatin-induced up-regulation of Notch1 at day 9 may be part of a feedback mechanism in response to Ngn3 up-regulation [32], [38] across stage 5, and may be opposed by the Notch1 functional antagonist Notch3 [38].

Ngn3, transiently expressed in committed precursors, is essential to their endocrine differentiation [3], [28] and involved in islet formation [48] and β-cell regeneration [33], [42]. Obestatin increased Ngn3 gene expression in MLCs at day 9 and reduced their proliferation, suggesting commitment toward the endocrine fate [31].

Interestingly, glucagon^+^ cells influence islet formation and fission during the second wave of Ngn3 up-regulation during pancreas ontogeny [49]. Obestatin induced glucagon expression at day 9. Simultaneously, ICC number was increased and ICC size decreased under obestatin treatment. Regardless of the shorter time, the above events recall those of islet fission, whereby an increase of glucagon^+^ cells at the sites of fission was noticed, followed by a transient reduction in islet size and increase in their number [49]. Therefore, obestatin-induced formation of equal-sized, more abundant and more differentiated ICCs compared to control, through increase in their number, initial size reduction and subsequent return to control size, is reminiscent of islet fission.

As glucagon^+^ cell proliferation is also involved in β-cell/pancreas regeneration [39], the above mechanisms may also participate to obestatin-induced pancreas regeneration [25], [39] and suggest a possible role in the endocrine pancreas maturation [50].

In summary, obestatin enhances *in vitro* generation of functional ICCs from islet-derived MLCs, and increases glucose-dependent C-peptide secretion. Whether these findings would be reproducible in human pancreatic islets, they may suggest implications in cell replacement therapy and highlight obestatin importance as therapeutic candidate in diabetes treatment. Moreover, obestatin modulation of FGFR/Notch/Ngn3 developmental pathways, combined to evidence of its early expression in the developing pancreas [50], suggests a role for this peptide in pancreas regeneration and formation.

## Supporting Information

Figure S1 **GPR39 and GLP-1R expression in control and obestatin-treated cultures.** (A) GPR39 and GLP-1R gene expression assessed by RT-PCR in enriched islets and in cultures at day 6 of stage 4. Results are representative of three independent experiments. Buffer alone was used as negative control (–). (B) Immunofluorescent staining of GPR39 and GLP-1R in MLCs in control and obestatin-treated cultures at the end of stage 4. Receptors are shown in green and nuclei in blue (DAPI). Each image is representative of three independent experiments (X40).
(EPS)

Figure S2 **Glucose-induced insulin secretion by ICCs, and insulin levels in cell culture medium.** (A) Insulin secretion assessed by RIA in ICCs stimulated with the indicated concentrations of glucose. Values are the mean ± SEM of three independent experiments (*P<0.05, **P<0.01 vs. control; ^##^ *P*<0.01 same condition vs. 0 mM glucose). (B) Insulin content measured in 300 µl of growth medium from control and obestatin-treated cultures, at the indicated time points. Insulin content was also assessed in the medium before being added to the cultures (Medium stage 4 and 5). Results are the mean ± SEM of three independent experiments (^#^ *P*<0.05 vs. day 9; ^##^ *P*<0.01 vs. day 6, n = 3).
(EPS)

Figure S3 **Obestatin inhibits FGF2 binding to MLCs at stage 5. FGF+MLCs were cultured in FGF-starved conditions, in either absence or presence of obestatin at the indicated days.** Graph shows the results expressed as percent of total cell number, that are the mean ± SEM of three independent experiments performed in duplicate (*P<0.05 vs. control). Right panel. Representative images (X20) of FGF2 staining (green) (n = 3); nuclei are shown in blue (DAPI).
(EPS)

Table S1 **Primers sequences, expected length of amplification products and PCR conditions (bp, base pair; mT, melting temperature).**
(EPS)

Table S2 **Enumeration of endocrine hormone-producing cells and BrdU+ cells (stage 5, day 9).**
(EPS)
