# Correction: Obestatin Enhances In Vitro Generation of Pancreatic Islets through Regulation of Developmental Pathways

**DOI:** 10.1371/annotation/070c66f7-8891-4ed8-9ef3-234dbf4e9148

**Published:** 2013-07-01

**Authors:** lessandra Baragli, Cristina Grande, Iacopo Gesmundo, Fabio Settanni, Marina Taliano, Davide Gallo, Eleonora Gargantini, Ezio Ghigo, Riccarda Granata

The name of the first author was spelled incorrectly. The correct name is: Alessandra Baragli. 

The correct citation is: Baragli A, Grande C, Gesmundo I, Settanni F, Taliano M, et al. (2013) Obestatin Enhances In Vitro Generation of Pancreatic Islets through Regulation of Developmental Pathways. PLoS ONE 8(5): e64374. doi:10.1371/journal.pone.0064374. The abbreviation in Contributions is correct.

